# Estimating financial and health burden by initial Medicare plan choice and history of cancer

**DOI:** 10.1093/haschl/qxaf001

**Published:** 2025-01-21

**Authors:** Shelley A Jazowski, Emma M Achola, Lauren Hersch Nicholas, William A Wood, Christopher R Friese, Stacie B Dusetzina

**Affiliations:** Department of Social Sciences and Health Policy, Wake Forest University School of Medicine, Winston-Salem, NC 27101, United States; Department of Health Policy, Vanderbilt University School of Medicine, Nashville, TN 37203, United States; Department of Health Policy, Vanderbilt University School of Medicine, Nashville, TN 37203, United States; Department of Medicine, Division of Geriatrics, University of Colorado Anschutz Medical Campus, Aurora, CO 80045, United States; University of Colorado Comprehensive Cancer Center, Aurora, CO 80045, United States; Department of Medicine, University of North Carolina at Chapel Hill School of Medicine, Chapel Hill, NC 27599, United States; Lineberger Comprehensive Cancer Center, University of North Carolina at Chapel Hill, Chapel Hill, NC 27599, United States; University of Michigan School of Nursing, Ann Arbor, MI 48109, United States; Department of Health Management and Policy, University of Michigan School of Public Health, Ann Arbor, MI 48109, United States; Rogel Cancer Center, University of Michigan, Ann Arbor, MI 48109, United States; Department of Health Policy, Vanderbilt University School of Medicine, Nashville, TN 37203, United States; Vanderbilt-Ingram Cancer Center, Nashville, TN 37232, United States

**Keywords:** Medicare, cancer, out-of-pocket spending, cost-related nonadherence, health status

## Abstract

Understanding the downstream consequences of initial Medicare plan selection is necessary to ensure access to and affordability of health care services, especially for older adults with serious illness. We used 2008-2020 data from the Health and Retirement Study to estimate financial and health burden by initial Medicare plan selection (traditional Medicare without supplemental coverage, traditional Medicare plus supplemental coverage, or Medicare Advantage) and self-reported history of cancer. Initially choosing benefits with greater financial protections (either traditional Medicare plus supplemental coverage or Medicare Advantage) relative to traditional Medicare without supplemental coverage was associated with lower levels of out-of-pocket spending and a lower likelihood of reporting cost-related medication nonadherence and fair or poor health. Policymakers should consider improving the adequacy of traditional Medicare coverage to ensure the affordability of health care services and reduce the burden of serious illness among older adults, especially those with a history of cancer.

## Introduction

When initially selecting Medicare benefits, older adults must not only weigh trade-offs^1^ between health care access and affordability but also manage strict enrollment deadlines that carry coverage or financial penalties.^2,3^ For example, individuals selecting Medicare Advantage must ensure their health care providers are in-network or they could encounter financial barriers when seeking out-of-network care,^4,5^ whereas those choosing traditional Medicare have 6 months to enroll in Medigap (to help reduce cost-sharing) or they could be charged high premiums or denied this supplemental coverage due to their health status.^3,5^ Initial enrollment decisions are further compounded by the number of plan options^6^ and limited availability of unbiased^7^ resources that provide timely, streamlined information regarding scope of benefits and cost-sharing estimates.^8,9^ Given the complexity of health coverage decisions, older adults often report feeling overwhelmed^8^ and select Medicare plans that are high-cost, low-value, or provide limited benefits.^10-13^

Consequently, selection of specific Medicare benefits has been associated with decreased access to and use of health services,^14-21^ increased issues with affording care,^22,23^ and poor clinical outcomes.^17,21,24,25^ Evidence suggests that individuals enrolled in traditional Medicare are less likely to report a usual source of care^14,15^ and receive preventive^15-17^ or primary care^16^ services than those enrolled in Medicare Advantage. Conversely, Medicare Advantage beneficiaries have lower rates of specialist visits,^18,19^ post-acute care,^20,21^ and hospital stays^19,26,27^ relative to their traditional Medicare counterparts. Some studies have found no difference in affordability of care by type of Medicare coverage;^14,26^ however, traditional Medicare beneficiaries without supplemental coverage frequently report problems paying medical bills or accessing necessary care due to the cost.^22,23^ In terms of health outcomes, traditional Medicare beneficiaries often have worse disease management,^21^ more hospital readmissions,^17,21,24^ and higher mortality rates^25^ compared to Medicare Advantage beneficiaries.

Understanding the implications of initial Medicare plan selection for beneficiaries with a history of cancer is especially important given the intensity and substantial cost of necessary care.^28-33^ In a prior study, we found that older adults with a history of cancer had a high likelihood of selecting traditional Medicare plus supplemental coverage, which may have been due to their prior experiences with navigating access barriers (eg, restrictive health care provider networks) and using high-cost health care services.^5^ While previous research has demonstrated differential utilization of cancer care services (eg, surgery and chemotherapy) and access to top-ranked cancer centers by type of Medicare coverage,^18,19,33^ the affordability of care and subsequent clinical outcomes associated with Medicare enrollment and a history of cancer is limited. Therefore, our objectives were to estimate financial and health burden by initial Medicare plan selection and self-reported history of cancer.

## Data and methods

### Study population

We used 2008-2020 data from the Health and Retirement Study, a nationally representative biennial survey of 43 000 US adults aged 50 years or older.^34^ We included respondents who were 65 or 66 years of age at initial Medicare plan selection, had no evidence of Medicare coverage in the 2 years prior to age eligibility, and completed at least 2 surveys (1 baseline survey to measure initial Medicare plan choice and history of cancer and at least 1 subsequent survey to measure financial and health burden) (Figure S1). We excluded individuals who were dually eligible for Medicaid or enrolled in Veterans Affairs or other military health plans at the time of initial Medicare enrollment (expected low out-of-pocket costs may influence their Medicare plan selection and subsequent financial burden).^5^

### Outcomes

Primary outcomes were identified in the survey waves following initial Medicare plan selection and included self-reported out-of-pocket spending, cost-related medication nonadherence, hospitalizations, and health status. Out-of-pocket spending included beneficiaries’ portion of costs for hospital stays, nursing home stays, outpatient surgeries, physician visits, dentist visits, home health care, special services (eg, outpatient rehabilitation services), and prescription medications. Out-of-pocket spending was inflation-adjusted to 2020 dollars using the Consumer Price Index for all Urban Consumers.^35,36^ Respondents who answered “Yes” to “Have you ended up taking less medication than was prescribed for you because of the cost?” were categorized as having experienced cost-related medication nonadherence. Beneficiaries were classified as having any hospitalization if they had “been a patient in the hospital overnight.” Finally, health status was dichotomized as reporting excellent, very good, or good health vs fair or poor health.^37^

### Independent variables

The main independent variables were self-reported initial Medicare plan selection and history of cancer. Medicare coverage was defined as choosing 1 of 3 mutually exclusive categories: traditional Medicare without supplemental coverage, traditional Medicare plus supplemental coverage (eg, Medigap and retiree benefits), or Medicare Advantage.^5^ Respondents with a history of cancer were identified based on their affirmative response to the following question at the time of initial Medicare plan selection: “Has a doctor ever told you that you have cancer or a malignant tumor, excluding minor skin cancer?”

### Covariates

Covariates included the following self-reported sociodemographic and health-related measures at time of initial Medicare plan selection: sex, race (White, Black, other), Hispanic ethnicity, married/partnered status, educational attainment (high school education or less vs above high school education), quartiles of wealth and assets (eg, real estate, investments, and bank accounts), census region, comorbidities (sum of diagnoses of hypertension, diabetes, stroke, arthritis, lung disease, heart condition, cognitive impairment, psychological or emotional issues),^5^ and smoking status.

### Statistical analysis

Baseline sociodemographic and health-related characteristics were compared between respondents with and without a history of cancer using chi-square tests. We used linear probability models and modified Poisson regression^38^ to assess the association between initial Medicare plan selection and our binary measures of cost-related medication nonadherence, any hospitalization, and health status. In addition, we used quantile regression to evaluate total out-of-pocket spending at the 25th, 50th, 75th, 90th, and 95th percentiles. All models adjusted for sociodemographic and health-related factors, used clustered standard errors to account for within-person correlation due to responses across multiple survey waves, and were stratified by a history of cancer. We present unweighted estimates because survey-weighted estimates may not be stable for measures of financial and health burden with a limited number of observations.^39,40^

### Sensitivity analysis

We conducted several sensitivity analyses to assess the robustness of our findings. First, some older adults covered by employer-sponsored plans may have delayed Medicare enrollment until retirement; therefore, we analyzed individuals who were 65 to 75 years of age at initial Medicare plan selection.^5^ Second, because an incident diagnosis of cancer could exacerbate financial burden and health outcomes, we excluded respondents who reported a diagnosis of cancer in the survey waves after initial Medicare plan selection. Third, we used an “intent-to-treat” approach in our primary analysis and ignored any plan switching that occurred after initial enrollment; thus, we excluded respondents who reported switching Medicare coverage in the survey wave immediately following initial plan selection. Fourth, since existing financial and health burden could influence future outcomes, we controlled for baseline measures of out-of-pocket spending, cost-related medication nonadherence, any hospitalization, and health status in our models. Last, high socioeconomic status, particularly financial resources, is more common among older adults enrolled in traditional Medicare plus supplemental coverage^41-43^ and thus, may minimize the independent association of initial coverage on measures of financial burden. As such, we did not adjust for quartiles of wealth and assets in models estimating cost-related medication nonadherence and out-of-pocket spending.

## Limitations

Our study had several limitations. First, due to the observational nature of this study, we were unable to establish a causal relationship between initial Medicare plan choice and financial and health burden. Second, data were self-reported and subject to associated biases (eg, recall); however, data collection methods minimize the extent of these biases^34^ and many variables have either been validated with claims data (eg, history of cancer) or demonstrated high validity when compared with other population-based surveys (eg, out-of-pocket spending).^44-46^ Third, we were unable to determine if and how health care provider networks and health service utilization impacted beneficiaries’ out-of-pocket spending. Fourth, we lacked information regarding prior health insurance coverage for respondents completing their first survey at 65 or 66 years of age and thus, may have misclassified some individuals with and without a history of cancer as newly enrolled in Medicare.^5^ Fifth, we were not able to discern the specific timing and stage of a cancer diagnosis and thus, could not evaluate the association between recency and severity of a diagnosis and our measures of financial and health burden. Sixth, we lacked data regarding financial literacy and the receipt of financial assistance (eg, cost-sharing support), which may have influenced our measures of financial burden. Seventh, our analysis focused on initial Medicare plan selection and subsequent financial and health burden. As such, future research is necessary to understand how access to, timeliness of, and quality of care differ by Medicare coverage and history of cancer. Eighth, due to the limited sample size of beneficiaries with a history of cancer, we may have lacked statistical power for some comparisons across types of Medicare coverage. Last, our study population was comprised of adults who aged into Medicare and may not represent the experiences of those who qualified for benefits due to end-stage renal disease or a disability.

## Results

### Cohort characteristics

A total of 3142 individuals aged 65 to 66 years old initially selected Medicare coverage from 2008 to 2018. Respondents completed a median of 3 surveys (IQR: 2, 4) after initial Medicare plan selection, which contributed to 9795 financial and health burden observations (cancer: 1177; noncancer: 8618).

At the time of initial Medicare enrollment, 12.35% of respondents reported a history of cancer (Table 1 and Table S1). The most common initial coverage type was traditional Medicare plus supplemental coverage (cancer: 59.54%; noncancer: 52.51%), followed by Medicare Advantage (cancer: 27.84%; noncancer: 27.31%), and traditional Medicare without supplemental coverage (cancer: 12.63%; noncancer: 20.19%). Sociodemographic and health-related characteristics were similar between respondents with and without a history of cancer. However, individuals with a history of cancer were less likely to be Black (12.63% vs 15.65%) or Hispanic (6.44% vs 10.06%), but more likely to have above a high school education (45.62% vs 37.04%) and report multiple comorbid conditions (63.66% vs 56.14%) relative to their counterparts without a history of cancer.

**Table 1.** Self-reported sociodemographic and health-related characteristics at time of initial Medicare plan selection.

	Study cohort (*n* = 3142)	Respondents with a history of cancer (*n* = 388)	Respondents without a history of cancer (*n* = 2754)	*P*-value
Initial plan type (*n*, %)				
Traditional Medicare without supplemental coverage	605 (19.26)	49 (12.63)	556 (20.19)	0.0013
Traditional Medicare plus supplemental coverage	1677 (53.37)	231 (59.54)	1446 (52.51)	
Medicare Advantage	860 (27.37)	108 (27.84)	752 (27.31)	
Sex (*n*, %)				
Male	1233 (39.24)	141 (36.34)	1092 (39.65)	0.2111
Female	1909 (60.76)	247 (63.66)	1662 (60.35)	
Race (*n*, %)				
White	2465 (78.45)	325 (83.76)	2140 (77.71)	0.0134
Black	480 (15.28)	49 (12.63)	431 (15.65)	
Other^a^	197 (6.27)	14 (3.61)	183 (6.64)	
Ethnicity (*n*, %)				
Non-Hispanic	2840 (90.39)	363 (93.56)	2477 (89.94)	0.0237
Hispanic	302 (9.61)	25 (6.44)	277 (10.06)	
Married or partnered (*n*, %)				
Yes	2330 (74.16)	296 (76.29)	2034 (73.86)	0.3055
No	812 (25.84)	92 (23.71)	720 (26.14)	
Education (*n*, %)				
High school or less	1945 (61.90)	211 (54.38)	1734 (62.96)	0.0011
Above high school	1197 (38.10)	177 (45.62)	1020 (37.04)	
Wealth^b^ (*n*, %)				
<$84 500	785 (24.98)	97 (25.00)	688 (24.98)	0.5936
$84 500−$293 000	787 (25.05)	90 (23.20)	697 (25.31)	
$293 001-$731 225	785 (24.98)	94 (24.23)	691 (25.09)	
>$731 225	785 (24.98)	107 (27.58)	678 (24.62)	
Geography (*n*, %)				
Northeast	417 (13.27)	52 (13.40)	365 (13.25)	0.2913
Midwest	801 (25.49)	86 (22.16)	715 (25.96)	
South	1308 (41.63)	163 (42.01)	1145 (41.58)	
West	616 (19.61)	87 (22.42)	529 (19.21)	
Comorbidities (*n*, %)				
0	465 (14.80)	39 (10.05)	426 (15.47)	0.0049
1	884 (28.13)	102 (26.29)	782 (28.40)	
≥2	1793 (57.07)	247 (63.66)	1546 (56.14)	
Current smoker (*n*, %)				
Yes	361 (11.49)	37 (9.54)	324 (11.76)	0.1975
No	2781 (88.51)	351 (90.46)	2430 (88.24)	

Source: Authors’ analysis of data from the Health and Retirement Study, 2008-2018.

^a^Other includes American Indian, Alaskan Native, Asian, Native Hawaiian, and Pacific Islander.

^b^Self-reported quartiles of wealth and assets were defined using the total wealth RAND variable (sum value of residences, vehicles, investments, bank accounts/savings less mortgages, loans, and debts).

### Initial Medicare plan selection and subsequent financial burden

Among individuals without a history of cancer, those initially choosing Medicare Advantage had statistically significant lower levels of out-of-pocket spending at the 50th (−$167.75), 75th (−$546.60), 90th (−$1132.29), and 95th (−$2362.26) percentiles than those enrolled in traditional Medicare without supplemental coverage (Table 2, Tables S2-S6). Differences in out-of-pocket spending were even more pronounced among respondents with a history of cancer. Out-of-pocket costs were $668.67, $1094.31, $2613.55, and $9884.27 less at the 50th, 75th, 90th, and 95th percentiles, respectively, for individuals initially enrolled in Medicare Advantage vs traditional Medicare without supplemental coverage. Moreover, for the highest spenders (90th and 95th percentiles) with a history of cancer, we observed statistically significant differences of $3195.03 and $9634.96 for those initially selecting traditional Medicare plus supplemental coverage compared to those choosing traditional Medicare without supplemental coverage.

**Table 2.** Association of initial Medicare plan selection with self-reported out-of-pocket spending.

	Study cohort	Respondents with a history of cancer	Respondents without a history of cancer
Adjusted estimates (95% confidence limits)^a,b,c^			
25th percentile			
Medicare Advantage	−$49.69 (−$120.12, $20.74)	−$303.78 (−$567.24, −$40.32)	−$28.22 (−$97.22, $40.78)
Traditional Medicare plus supplemental coverage	$31.18 (−$32.24, $94.61)	−$139.07 (−$382.88, $104.75)	$54.75 (−$7.14, $116.64)
Traditional Medicare w/o supplemental coverage	Ref	Ref	Ref
50th percentile			
Medicare Advantage	−$200.96 (−$344.81, −$57.10)	−$668.67 (−$1177.90, −$159.44)	−$167.75 (−$314.74, −$20.75)
Traditional Medicare plus supplemental coverage	$45.37 (−$84.17, $174.92)	−$232.63 (−$703.89, $238.63)	$86.18 (−$45.67, $218.03)
Traditional Medicare w/o supplemental coverage	Ref	Ref	Ref
75th percentile			
Medicare Advantage	−$565.98 (−$862.93, −$269.03)	−$1094.31 (−$2053.67, −$134.96)	−$546.60 (−$859.60, −$233.59)
Traditional Medicare plus supplemental coverage	$10.02 (−$257.38, $277.43)	−$875.93 (−$1763.74, $11.89)	$53.95 (−$226.80, $334.70)
Traditional Medicare w/o supplemental coverage	Ref	Ref	Ref
90th percentile			
Medicare Advantage	−$1306.44 (−$2049.10, −$563.78)	−$2613.55 (−$5173.95, −$53.16)	−$1132.29 (−$1936.98, −$327.60)
Traditional Medicare plus supplemental coverage	−$467.01 (−$1135.78, $201.76)	−$3195.03 (−$5564.50, −$825.56)	−$293.46 (−$1015.23, $428.30)
Traditional Medicare w/o supplemental coverage	Ref	Ref	Ref
95th percentile			
Medicare Advantage	−$2479.00 (−$3721.14, −$1236.86)	−$9884.27 (−$13 383.85, −$6384.70)	−$2362.26 (−$3593.39, −$1131.14)
Traditional Medicare plus supplemental coverage	−$790.12 (−$1908.67, $328.43)	−$9634.96 (−$12 873.59, −$6396.34)	−$452.92 (−$1557.17, $651.34)
Traditional Medicare w/o supplemental coverage	Ref	Ref	Ref

Source: Authors’ analysis of data from the Health and Retirement Study, 2008-2020.

^a^A total of 9795, 1177, and 8618 observations were included in the models of the study cohort, respondents with a history of cancer, and respondents without a history of cancer, respectively. Full model results are presented in Tables S1-S5.

^b^Out-of-pocket spending was measured in the survey waves following initial Medicare plan selection and included beneficiaries’ portion of costs for hospital stays, nursing home stays, outpatient surgeries, physician visits, dentist visits, home health care, special services, and prescription medications. Out-of-pocket spending was inflation-adjusted to 2020 dollars using the Consumer Price Index for all Urban Consumers.

^c^Out-of-pocket spending at the 25th, 50th, 75th, 90th, and 95th percentiles was estimated with quantile regression. All models were adjusted for baseline sociodemographic and health-related characteristics.

**Figure 1.**
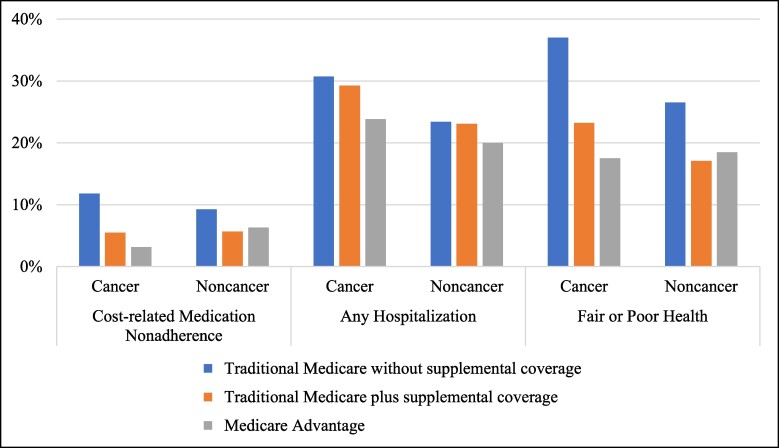
Self-reported financial and health burden by initial Medicare plan selection and history of cancer. Source: Authors’ analysis of data from the Health and Retirement Study, 2008-2020. ^a^Figure represents pooled person time observations.

Reports of cost-related medication nonadherence were low (cancer: 5.54%; noncancer: 6.53%) and most often occurred among respondents who initially chose traditional Medicare without supplemental coverage (cancer: 11.81%; noncancer: 9.25%; Figure 1). Initial Medicare plan selection was not associated with experiencing cost-related medication nonadherence among individuals without a history of cancer (Table 3, Table S7). Conversely, individuals with a history of cancer who initially selected Medicare Advantage had a lower probability of reporting cost-related medication nonadherence (adjusted risk difference [aRD] −0.07, 95% confidence limit [CL] −0.13, −0.01; adjusted risk ratio [aRR] 0.33, 95% CL 0.14, 0.78) compared to those who chose traditional Medicare without supplemental coverage.

**Table 3.** Association of initial Medicare plan selection with self-reported financial and health burden.

	Adjusted risk differences (95% confidence limits)^a,b^			Adjusted risk ratios (95% confidence limits)^a,b^		
	Study cohort	Respondents with a history of cancer	Respondents without a history of cancer	Study cohort	Respondents with a history of cancer	Respondents without a history of cancer
Cost-related medication nonadherence^c^						
Medicare Advantage	−0.02 (−0.04, −0.001)	−0.07 (−0.13, −0.01)	−0.02 (−0.04, 0.01)	0.74 (0.56, 0.97)	0.33 (0.14, 0.78)	0.79 (0.59, 1.06)
Traditional Medicare plus supplemental coverage	−0.02 (−0.04, 0.003)	−0.05 (−0.11, 0.02)	−0.01 (−0.04, 0.01)	0.81 (0.62, 1.04)	0.58 (0.29, 1.16)	0.82 (0.63, 1.08)
Traditional Medicare w/o supplemental coverage	Ref	Ref	Ref	Ref	Ref	Ref
Any hospitalization^c^						
Medicare Advantage	−0.02 (−0.06, 0.01)	−0.02 (−0.12, 0.09)	−0.02 (−0.06, 0.01)	0.89 (0.78, 1.03)	0.93 (0.65, 1.32)	0.89 (0.77, 1.04)
Traditional Medicare plus supplemental coverage	0.004 (−0.02, 0.03)	0.03 (−0.07, 0.13)	0.002 (−0.03, 0.03)	1.02 (0.90, 1.14)	1.11 (0.82, 1.52)	1.01 (0.89, 1.15)
Traditional Medicare w/o supplemental coverage	Ref	Ref	Ref	Ref	Ref	Ref
Fair or poor health status^c^						
Medicare Advantage	−0.07 (−0.10, −0.03)	−0.15 (−0.29, −0.01)	−0.06 (−0.10, −0.02)	0.75 (0.64, 0.88)	0.60 (0.39, 0.93)	0.78 (0.66, 0.92)
Traditional Medicare plus supplemental coverage	−0.05 (−0.08, −0.01)	−0.09 (−0.22, 0.05)	−0.04 (−0.08, −0.01)	0.83 (0.72, 0.95)	0.79 (0.55, 1.15)	0.83 (0.72, 0.97)
Traditional Medicare w/o supplemental coverage	Ref	Ref	Ref	Ref	Ref	Ref

Source: Authors’ analysis of data from the Health and Retirement Study, 2008-2020.

^a^Financial and health burden were measured in the survey waves following initial Medicare plan selection. A total of 9795, 1177, and 8618 observations were included in the models of the study cohort, respondents with a history of cancer, and respondents without a history of cancer, respectively. Full model results are presented in Tables S6-S8.

^b^Risk differences were estimated with linear probability models, and risk ratios were estimated with modified Poisson regression models. All models were adjusted for baseline sociodemographic and health-related characteristics, and standard errors were clustered at the respondent-level to account for repeated measures.

^c^Missing observations were excluded from the models. Specifically, 40 (4 cancer; 36 noncancer) observations were excluded from the cost-related medication nonadherence model, 37 (cancer: 3; noncancer 34) observations were excluded from the hospitalizations model, and 8 (2 cancer; 6 noncancer) were excluded from the health status model.

### Initial Medicare plan selection and subsequent health burden

Respondents without a history of cancer were less likely to report any hospitalization compared to those with a history of cancer (22.32% vs 27.94%). Among individuals without a history of cancer, reports of any hospitalization were less common for those initially enrolled in Medicare Advantage (19.99%) and similar for those selecting traditional Medicare plus and without supplemental coverage (23.10% and 23.41%, respectively; Figure 1). Reporting of any hospitalization by specific type of Medicare coverage was similar for respondents with a history of cancer. Initial Medicare plan selection was not associated with the reporting of any hospitalization in either individuals with or without a history of cancer (Table 3, Table S8).

Self-reported fair or poor health was less common among beneficiaries without a history of cancer relative to those with a history of cancer (19.31% vs 23.14%). Reports of fair or poor health were the highest for respondents with and without a history of cancer who initially selected traditional Medicare without supplemental coverage (37.01% and 26.53%, respectively; Figure 1). Among individuals without a history of cancer, those initially enrolled in Medicare Advantage or traditional Medicare plus supplemental coverage had a lower likelihood (aRD −0.06, 95% CL −0.10, −0.02; aRR 0.78, 95% CL 0.66, 0.92 and aRD −0.04, 95% CL −0.08, −0.01; aRR 0.83, 95% CL 0.72, 0.97, respectively) of reporting fair or poor health compared to their counterparts enrolled in traditional Medicare without supplemental coverage (Table 3, Table S9). Findings were similar for individuals with a history of cancer, although the difference in health status between those enrolled in traditional Medicare plus and without supplemental coverage was not statistically significant.

### Sensitivity analyses

When quartiles of wealth and assets were excluded from models estimating financial burden, Medicare benefits with greater financial protections (either traditional Medicare plus supplemental coverage or Medicare Advantage) were associated with a lower likelihood of reporting cost-related medication nonadherence for respondents with and without a history of cancer (Table S10). For example, respondents with and without a history of cancer who initially selected traditional Medicare plus supplemental coverage had a 49% (95% CL 0.26, 0.98) and 29% (95% CL 0.54, 0.94) lower probability of reporting cost-related medication nonadherence, respectively, compared to their counterparts who chose traditional Medicare without supplemental coverage.

In analyses that expanded beneficiaries’ ages at initial plan selection (65 to 75 years of age), excluded beneficiaries who reported a cancer diagnosis after initial Medicare plan selection, excluded beneficiaries who reported switching Medicare coverage in the survey wave immediately following initial plan selection, and controlled for baseline measures of out-of-pocket spending, cost-related medication nonadherence, any hospitalization, and health status, findings were consistent with our primary analyses and are shown in Tables S10-S15.

## Discussion

In this study that assessed financial and health burden by initial Medicare plan selection and history of cancer, we observed lower levels of out-of-pocket spending among older adults enrolled in Medicare Advantage vs traditional Medicare without supplemental coverage. Our findings align with previous studies that have demonstrated an association between Medicare Advantage enrollment and lower health care expenditures, which could be due to cost-effective contracts with health care providers,^18,19,47^ decreased health service utilization,^18,27,47^ and capping beneficiaries’ in-network costs.^6^ Lower levels of out-of-pocket spending for individuals with and without a history of cancer may be at risk as hospitals, health systems, and physician practices end contracts with Medicare Advantage plans.^48,49^ Future research is needed to understand how Medicare Advantage contract terminations impact plan selection and health care affordability, especially among individuals without out-of-network coverage or maximums.

We also found that beneficiaries with a history of cancer who initially chose traditional Medicare plus supplemental coverage had lower levels of out-of-pocket spending. Prior experiences with high-cost health services may have prompted individuals with a history of cancer to select wraparound coverage,^5^ which in turn, likely helped with cost-sharing for hospital and medical services.^50^ Given that traditional Medicare beneficiaries without supplemental coverage are responsible for 20% of cancer-related expenditures, ranging from $43 500 in the year following diagnosis to $109 700 in the last year of life,^51^ state governments should consider expanding consumer protections for supplemental or Medigap plans. For example, extending guaranteed issue protections beyond the initial open enrollment period (eg, annually or continuously)^3^ and implementing community rating for premiums^3^ would ensure that beneficiaries diagnosed with a new or pre-existing condition, such as cancer, are not denied and able to afford supplemental coverage.

Consistent with prior research,^52^ we found that individuals with a history of cancer who were enrolled in Medicare Advantage were less likely to report cost-related medication nonadherence relative to those who selected traditional Medicare without supplemental coverage. One potential explanation for this finding is that prior authorization, step therapy, or other utilization management strategies^53^ may have influenced the prescribing of less costly medications for beneficiaries who were actively treated for or managing the long-term effects of cancer.^32,54^ Moreover, Medicare Advantage health and drug plans are rated on quality metrics (eg, care coordination and medication management)^55^ and employ methods to monitor and incentivize prescribing behavior,^53^ both of which could have prompted the use of lower cost treatments. Another possible explanation is that Medicare Advantage plans cover core cancer-related benefits, including care management and navigation,^56^ that may have facilitated access to financial support to aid with prescription drug costs.

Last, we observed an inverse association between robust coverage and self-reported fair or poor health status. Beneficiaries who initially selected traditional Medicare plus supplemental coverage or Medicare Advantage may have been disproportionately “healthier”^57-59^ compared to their counterparts who enrolled in traditional Medicare without supplemental coverage. For example, Medicare Advantage plans have historically attracted low-risk individuals (eg, fewer health conditions and less costly than their peers enrolled in traditional Medicare without supplemental coverage),^57^ although recent changes to Medicare policies (eg, longer lock-in periods) have decreased this favorable selection over time.^57^ Increasing out-of-pocket burden may also have contributed to the high likelihood of reporting fair or poor health among beneficiaries enrolled in traditional Medicare without supplemental coverage. Specifically, out-of-pocket burden has been associated with poor clinical outcomes, mental health, and health-related quality of life.^28,60,61^

Our findings illustrate the potential benefits of adequate insurance coverage and highlight how changes to the design of traditional Medicare could support the 3.2 million beneficiaries without supplemental coverage.^62^ While provisions of the Inflation Reduction Act (eg, $2000 out-of-pocket limit)^63^ could reduce financial burden and nonadherence associated with high-cost prescription medications covered by Medicare Part D, the Centers for Medicare & Medicaid Services (CMS) and policymakers should focus on increasing financial protections for hospital (Part A) and medical (Part B) benefits. For example, implementing an out-of-pocket maximum that is equivalent to that in Medicare Advantage^64,65^ could not only reduce financial burden among beneficiaries with the highest out-of-pocket spending and/or substantial health needs but also further address favorable selection in Medicare Advantage plans.^64,65^ In addition, CMS and policymakers should consider expanding eligibility for and comprehensiveness of Medicare Savings Programs. Although CMS has streamlined enrollment processes for these programs (eg, Social Security Income recipients are automatically enrolled in the Qualified Medicare Beneficiary Program),^66^ eliminating asset tests and further subsidizing beneficiary cost-sharing could potentially minimize the financial and disease burden of the most vulnerable older adults,^67^ especially those with a history of cancer.

## Conclusion

In this cohort study of Medicare beneficiaries with and without a history of cancer, we found that those initially enrolled in plans with greater financial protections (eg, out-of-pocket maximums and supplemental coverage) had lower levels of out-of-pocket spending and a lower likelihood of reporting fair or poor health compared with their counterparts enrolled in traditional Medicare without supplemental coverage. Policymakers should consider improving the adequacy of traditional Medicare coverage, including the implementation of out-of-pocket limits and the expansion of eligibility for cost-sharing subsidies for hospital and medical care, to ensure the affordability of health care services and reduce the burden of serious illness among older adults.

## Supplementary Material

qxaf001_Supplementary_Data
