# Supplementary material for: Estimating financial and health burden by initial Medicare plan choice and history of cancer
Source: Health Aff Sch. 2025 Jan 21;3(1):qxaf001. doi: 10.1093/haschl/qxaf001 (PMC11747364; doi:10.1093/haschl/qxaf001)
Supplement: qxaf001_Supplementary_Data [file qxaf001_supplementary_data.zip › Supplement_121624_Clean.docx]

**Supplement**

**Figure S1.** Study Flow Diagram

**Table S1.** Self-reported Sociodemographic and Health-related Characteristics by Initial Medicare Plan Selection

**Table S2.** Multivariable Model Assessing the Association of Initial Medicare Plan Choice with Self-reported Out-of-Pocket Spending at the 25^th^ Percentile

**Table S3.** Multivariable Model Assessing the Association of Initial Medicare Plan Choice with Self-reported Out-of-Pocket Spending at the 50^th^ Percentile

**Table S4.** Multivariable Model Assessing the Association of Initial Medicare Plan Choice with Self-reported Out-of-Pocket Spending at the 75^th^ Percentile

**Table S5.** Multivariable Model Assessing the Association of Initial Medicare Plan Choice with Self-reported Out-of-Pocket Spending at the 90^th^ Percentile

**Table S6.** Multivariable Model Assessing the Association of Initial Medicare Plan Choice with Self-reported Out-of-Pocket Spending at the 95^th^ Percentile

**Table S7.** Multivariable Model Assessing the Association of Initial Medicare Plan Choice with Self-reported Cost-related Medication Nonadherence

**Table S8.** Multivariable Model Assessing the Association of Initial Medicare Plan Choice with Any Self-reported Overnight Hospital Stay

**Table S9.** Multivariable Model Assessing the Association of Initial Medicare Plan Choice with Self-reported Fair or Poor Health Status

**Table S10.** Sensitivity Analysis Assessing the Association of Initial Medicare Plan Choice with Self-reported Financial and Health Burden

**Table S11.** Sensitivity Analysis Assessing the Association of Initial Medicare Plan Selection with Self-reported Out-of-Pocket Spending (Among Beneficiaries Aged 65-75 at Initial Plan Selection)

**Table S12.** Sensitivity Analysis Assessing the Association of Initial Medicare Plan Selection with Self-reported Out-of-Pocket Spending (Excluding Beneficiaries with a Cancer Diagnosis After Initial Plan Selection)

**Table S13.** Sensitivity Analysis Assessing the Association of Initial Medicare Plan Selection with Self-reported Out-of-Pocket Spending (Excluding Beneficiaries who Switched Medicare Coverage After Initial Selection)

**Table S14.** Sensitivity Analysis Assessing the Association of Initial Medicare Plan Selection with Self-reported Out-of-Pocket Spending (Adjusted for Baseline Out-of-Pocket Spending)

**Table S15.** Sensitivity Analysis Assessing the Association of Initial Medicare Plan Selection with Self-reported Out-of-Pocket Spending (Removed Wealth and Assets from the Model)

**Figure S1.** Study Flow Diagram

HRS participants

(n=43,559)

Excluded (n=22,867)

- Died prior to 2008 (n=8,551)
- Member of overlapping households (n=8)
- Without Medicare coverage (n=14,308)

HRS participants with Medicare

(n=20,692)

Excluded (n=17,550)

- Not first interview or without a 2-year lookback period (n=1,296)
- Prior Medicare coverage (9,964)
- Aged <65 years of age at initial Medicare plan selection (n=3,628)
- Aged >66 years of age at initial Medicare plan selection (n=1,396)
- Medicaid dual-eligibles, Veterans Affairs/military coverage or did not select mutually exclusive coverage type (n=597)
- First survey completed in 2020 (n=447)
- Did not complete a subsequent survey (n=222)

Study Cohort

(n=3,142)

**Source:** Authors’ analysis of data from the Health and Retirement Study, 2008-2020.

**Table S1.** Self-reported Sociodemographic and Health-related Characteristics by Initial Medicare Plan Selection

|  | **Traditional Medicare without Supplemental Coverage**  **(n=605)** | **Traditional Medicare Plus Supplemental Coverage**  **(n=1677)** | **Medicare Advantage (n=860)** | **P Value** |
| --- | --- | --- | --- | --- |
| History of cancer (n, %) |  |  |  |  |
| Yes | 49 (8.10) | 231 (13.77) | 108 (12.56) | 0.0013 |
| No | 556 (91.90) | 1446 (86.23) | 752 (87.44) |  |
| Sex (n, %) |  |  |  |  |
| Male | 262 (43.31) | 653 (38.94) | 318 (36.98) | 0.0472 |
| Female | 343 (56.69) | 1042 (61.06) | 542 (63.02) |  |
| Race (n, %) |  |  |  |  |
| White | 416 (68.76) | 1409 (84.02) | 640 (74.42) | <0.0001 |
| Black | 129 (21.32) | 193 (11.51) | 158 (18.37) |  |
| Other^a^ | 60 (9.92) | 75 (4.47) | 62 (7.21) |  |
| Ethnicity (n, %) |  |  |  |  |
| Non-Hispanic | 509 (84.13) | 1585 (94.51) | 746 (86.74) | <0.0001 |
| Hispanic | 96 (15.87) | 92 (5.49) | 114 (13.26) |  |
| Married or partnered (n,%) |  |  |  |  |
| Yes | 379 (62.64) | 1313 (78.29) | 638 (74.19) | <0.0001 |
| No | 226 (37.36) | 364 (21.71) | 222 (25.81) |  |
| Education (n, %) |  |  |  |  |
| High school or less | 441 (72.89) | 940 (56.05) | 564 (65.58) | <0.0001 |
| Above high school | 164 (27.11) | 737 (43.95) | 296 (34.42) |  |
| Wealth^a^ (n, %) |  |  |  |  |
| <$84,500 | 252 (41.65) | 295 (17.59) | 238 (27.67) | <0.0001 |
| $84,500-$293,000 | 164 (27.11) | 399 (23.79) | 224 (26.05) |  |
| $293,001-$731,225 | 92 (15.21) | 488 (29.10) | 205 (23.84) |  |
| >$731,225 | 97 (16.03) | 495 (29.52) | 193 (22.44) |  |
| Geography (n, %) |  |  |  |  |
| Northeast | 65 (10.74) | 256 (15.27) | 96 (11.16) | <0.0001 |
| Midwest | 135 (22.31) | 460 (27.43) | 206 (23.95) |  |
| South | 301 (49.75) | 679 (40.49) | 328 (38.14) |  |
| West | 104 (17.19) | 282 (16.82) | 230 (26.74) |  |
| Comorbidities (n, %) |  |  |  |  |
| 0 | 92 (15.21) | 246 (14.67) | 127 (14.77) | 0.9881 |
| 1 | 170 (28.10) | 477 (28.44) | 237 (27.56) |  |
| ≥2 | 343 (56.69) | 954 (56.89) | 496 (57.67) |  |
| Current smoker (n, %) |  |  |  |  |
| Yes | 99 (16.36) | 169 (10.08) | 93 (10.81) | 0.0001 |
| No | 506 (83.64) | 1508 (89.92) | 767 (89.19) |  |

**Source:** Authors’ analysis of data from the Health and Retirement Study, 2008-2018.

^a^ Other includes American Indian, Alaskan Native, Asian, Native Hawaiian, Pacific Islander.

^b^ Self-reported quartiles of wealth and assets were defined using the total wealth RAND variable (sum value of residences, vehicles, investments, bank accounts/savings less mortgages, loans, and debts).

**Table S2.** Multivariable Model Assessing the Association of Initial Medicare Plan Choice with Self-reported Out-of-Pocket Spending at the 25^th^ Percentile

|  | **Respondents with a History of Cancer** | **Respondents without a History of Cancer** |
| --- | --- | --- |
|  | **Adjusted Estimates (95% Confidence Limits)**^a,b^ | |
| Intercept | 467.20 (-34.33, 968.74) | 412.50 (269.30, 555.70) |
| Initial plan type |  |  |
| Medicare Advantage | -303.78 (-567.24, -40.32) | -28.22 (-97.22, 40.78) |
| Traditional Medicare plus supplemental coverage | -139.07 (-382.88, 104.75) | 54.75 (-7.14, 116.64) |
| Traditional Medicare without supplemental coverage | Ref | Ref |
| Sex |  |  |
| Male | Ref | Ref |
| Female | 198.51 (42.23, 354.79) | 105.13 (57.52, 152.74) |
| Race |  |  |
| White | Ref | Ref |
| Black | -300.95 (-538.65, -63.25) | -230.92 (-299.80, -162.04) |
| Other | -659.24 (-1106.00, -212.49) | -93.31 (-199.29, 12.67) |
| Ethnicity |  |  |
| Non-Hispanic | Ref | Ref |
| Hispanic | 239.30 (-108.91, 587.50) | 229.65 (139.69, 319.61) |
| Married or partnered |  |  |
| Yes | -128.57 (-308.75, 51.61) | -14.21 (-70.23, 41.82) |
| No | Ref | Ref |
| Education |  |  |
| High school or less | Ref | Ref |
| Above high school | 313.40 (162.00, 464.80) | 251.48 (199.90, 303.06) |
| Wealth |  |  |
| <$84,500 | -151.53 (-371.48, 68.42) | -209.57 (-286.20, -132.94) |
| $84,500-$293,000 | 68.89 (-142.13, 279.90) | -130.54 (-199.13, -61.95) |
| $293,001-$731,225 | 77.84 (-117.75, 273.44) | -72.07 (-137.09, -7.05) |
| >$731,225 | Ref | Ref |
| Geography |  |  |
| Northeast | -289.35 (-536.25, -42.45) | -43.72 (-125.48, 38.03) |
| Midwest | 39.86 (-175.00, 254.72) | 4.27 (-67.30, 75.85) |
| South | -325.29 (-521.06, -129.52) | -3.43 (-68.97, 62.10) |
| West | Ref | Ref |
| Comorbidities |  |  |
| 0 | -588.01 (-826.80, -349.23) | -414.87 (-480.62, -349.11) |
| 1 | -304.95 (-473.17, -136.73) | -317.14 (-369.97, -264.32) |
| ≥2 | Ref | Ref |
| Current smoker |  |  |
| Yes | Ref | Ref |
| No | 356.18 (98.79, 613.58) | 88.10 (15.89, 160.30) |

**Source:** Authors’ analysis of data from the Health and Retirement Study, 2008-2020.

^a^ Out-of-pocket spending was measured in the survey waves following initial Medicare plan selection and included beneficiaries’ portion of costs for hospital stays, nursing home stays, outpatient surgeries, physician visits, dentist visits, home health care, special services, and prescription medications. Outcomes were pooled and 1,177 and 8,618 observations were included in the models of respondents with and without a history of cancer, respectively.

^b^ Out-of-pocket spending was inflation-adjusted to 2020 dollars using the Consumer Price Index for all Urban Consumers.

**Table S3.** Multivariable Model Assessing the Association of Initial Medicare Plan Choice with Self-reported Out-of-Pocket Spending at the 50^th^ Percentile

|  | **Respondents with a History of Cancer** | **Respondents without a History of Cancer** |
| --- | --- | --- |
|  | **Adjusted Estimates (95% Confidence Limits)**^a,b^ | |
| Intercept | 1304.59 (335.21, 2273.97) | 1539.24 (1234.15, 1844.32) |
| Initial plan type |  |  |
| Medicare Advantage | -668.67 (-1177.90, -159.44) | -167.75 (-314.74, -20.75) |
| Traditional Medicare plus supplemental coverage | -232.63 (-703.89, 238.63) | 86.18 (-45.67, 218.03) |
| Traditional Medicare without supplemental coverage | Ref | Ref |
| Sex |  |  |
| Male | Ref | Ref |
| Female | 286.17 (-15.90, 588.24) | 222.20 (120.25, 324.15) |
| Race |  |  |
| White | Ref | Ref |
| Black | -458.02 (-917.44, 1.41) | -363.78 (-510.53, -217.03) |
| Other | 99.82 (-763.69, 963.32) | -67.55 (-29335, 158.24) |
| Ethnicity |  |  |
| Non-Hispanic | 1119.82 (446.79, 1792.84) | 331.61 (139.95, 523.27) |
| Hispanic | Ref | Ref |
| Married or partnered |  |  |
| Yes | Ref | Ref |
| No | -45.54 (-393.80, 302.71) | -27.04 (-146.40, 92.33) |
| Education |  |  |
| High school or less | Ref | Ref |
| Above high school | 239.50 (-53.13, 532.14) | 360.04 (250.15, 469.93) |
| Wealth |  |  |
| <$84,500 | -230.62 (-655.74, 194.50) | -477.65 (-640.91, -314.38) |
| $84,500-$293,000 | -172.22 (-580.08, 235.64) | -180.32 (-326.46, -34.19) |
| $293,001-$731,225 | 535.92 (157.87, 913.97) | -147.34 (-285.88, -8.81) |
| >$731,225 | Ref | Ref |
| Geography |  |  |
| Northeast | -929.95 (-1407.16, -452.75) | -179.80 (-353.98, -5.63) |
| Midwest | 190.28 (-225.01, 605.56) | -67.32 (-219.81, 85.17) |
| South | -437.50 (-815.89, -59.11) | -45.12 (-184.74, 94.49) |
| West | Ref | Ref |
| Comorbidities |  |  |
| 0 | -1110.13 (-1571.66, -648.60) | -956.25 (-1096.34, -816.15) |
| 1 | -743.44 (-1068.59, -418.29) | -781.16 (-893.70, -668.63) |
| ≥2 | Ref | Ref |
| Current smoker |  |  |
| Yes | Ref | Ref |
| No | 235.71 (-261.79, 733.21) | 142.02 (-11.82, 295.86) |

**Source:** Authors analysis of data from the Health and Retirement Study, 2008-2020.

^a^ Out-of-pocket spending was measured in the survey waves following initial Medicare plan selection and included beneficiaries’ portion of costs for hospital stays, nursing home stays, outpatient surgeries, physician visits, dentist visits, home health care, special services, and prescription medications. Outcomes were pooled and 1,177 and 8,618 observations were included in the models of respondents with and without a history of cancer, respectively.

^b^ Out-of-pocket spending was inflation-adjusted to 2020 dollars using the Consumer Price Index for all Urban Consumers.

**Table S4.** Multivariable Model Assessing the Association of Initial Medicare Plan Choice with Self-reported Out-of-Pocket Spending at the 75^th^ Percentile

|  | **Respondents with a History of Cancer** | **Respondents without a History of Cancer** |
| --- | --- | --- |
|  | **Adjusted Estimates (95% Confidence Limits)**^a,b^ | |
| Intercept | 3350.29 (1524.04, 5176.54) | 5029.01 (4379.38, 5678.63) |
| Initial plan type |  |  |
| Medicare Advantage | -1094.31 (-2053.67, -134.96) | -546.60 (-859.60, -233.59) |
| Traditional Medicare plus supplemental coverage | -875.93 (-1763.75, 11.89) | 53.95 (-226.80, 334.70) |
| Traditional Medicare without supplemental coverage | Ref | Ref |
| Sex |  |  |
| Male | Ref | Ref |
| Female | 156.07 (-413.01, 725.15) | 224.97 (7.89, 442.06) |
| Race |  |  |
| White | Ref | Ref |
| Black | -428.01 (-1293.54, 437.53) | -652.01 (-964.48, -339.53) |
| Other | 554.94 (-1071.84, 2181.72) | 43.99 (-436.80, 524.77) |
| Ethnicity |  |  |
| Non-Hispanic | 2369.70 (1101.86, 3637.73) | 216.68 (-191.42, 624.77) |
| Hispanic | Ref | Ref |
| Married or partnered |  |  |
| Yes | Ref | Ref |
| No | -252.22 (-908.30, 403.87) | -72.43 (-326.59, 181.74) |
| Education |  |  |
| High school or less | Ref | Ref |
| Above high school | 343.93 (-207.37, 895.23) | 476.66 (242.67, 710.64) |
| Wealth |  |  |
| <$84,500 | -396.31 (-1197.22, 405.59) | -119.87 (-1467.52, -772.23) |
| $84,500-$293,000 | -329.61 (-1097.99, 438.78) | -653.96 (-965.12, -342.79) |
| $293,001-$731,225 | 819.25 (107.02, 1531.48) | -412.68 (-707.66, -117.69) |
| >$731,225 | Ref | Ref |
| Geography |  |  |
| Northeast | -1468.81 (-2367.84, -569.78) | -574.61 (-945.48, -203.74) |
| Midwest | 195.58 (-586.79, 977.95) | -405.61 (-730.31, -80.91) |
| South | -420.66 (-1133.52, 292.20) | -289.77 (-587.06, 7.52) |
| West | Ref | Ref |
| Comorbidities |  |  |
| 0 | -2082.22 (-2897.72, -1158.73) | -2035.58 (-2333.88, -1737.28) |
| 1 | -886.68 (-1499.23, -274.12) | -1616.68 (-1856.31, -1377.06) |
| ≥2 | Ref | Ref |
| Current smoker |  |  |
| Yes | Ref | Ref |
| No | -315.20 (-1252.46, 622.06) | -136.04 (-463.61, 191.53) |

**Source:** Authors analysis of data from the Health and Retirement Study, 2008-2020.

^a^ Out-of-pocket spending was measured in the survey waves following initial Medicare plan selection and included beneficiaries’ portion of costs for hospital stays, nursing home stays, outpatient surgeries, physician visits, dentist visits, home health care, special services, and prescription medications. Outcomes were pooled and 1,177 and 8,618 observations were included in the models of respondents with and without a history of cancer, respectively.

^b^ Out-of-pocket spending was inflation-adjusted to 2020 dollars using the Consumer Price Index for all Urban Consumers.

**Table S5.** Multivariable Model Assessing the Association of Initial Medicare Plan Choice with Self-reported Out-of-Pocket Spending at the 90^th^ Percentile

|  | **Respondents with a History of Cancer** | **Respondents without a History of Cancer** |
| --- | --- | --- |
|  | **Adjusted Estimates (95% Confidence Limits)**^a,b^ | |
| Intercept | 7098.56 (2224.54, 11972.59) | 9947.09 (8277.00, 11617.19) |
| Initial plan type |  |  |
| Medicare Advantage | -2613.55 (-5173.95, -53.16) | -1132.29 (-1936.98, -327.60) |
| Traditional Medicare plus supplemental coverage | -3195.03 (5564.50, -825.56) | -293.46 (-1015.23, 428.30) |
| Traditional Medicare without supplemental coverage | Ref | Ref |
| Sex |  |  |
| Male | Ref | Ref |
| Female | 711.53 (-807.27, 2230.33) | 334.06 (-224.02, 892.14) |
| Race |  |  |
| White | Ref | Ref |
| Black | -1972.86 (-4282.86, 337.12) | -986.75 (-1790.07, -183.43) |
| Other | 904.43 (-3437.25, 5246.10) | 1565.62 (329.59, 2801.64) |
| Ethnicity |  |  |
| Non-Hispanic | 4542.40 (1158.45, 7926.34) | 365.83 (-683.34, 1415.00) |
| Hispanic | Ref | Ref |
| Married or partnered |  |  |
| Yes | Ref | Ref |
| No | 368.91 (-1382.11, 2119.92) | 44.31 (-609.11, 697.74) |
| Education |  |  |
| High school or less | Ref | Ref |
| Above high school | -329.02 (-1800.38, 1142.33) | 601.17 (-0.37, 1202.72) |
| Wealth |  |  |
| <$84,500 | -676.11 (-2813.62, 1461.41) | -2262.89 (-3156.63, -1369.16) |
| $84,500-$293,000 | -476.05 (-2526.76, 1574.66) | -1052.08 (-1852.04, -252.11) |
| $293,001-$731,225 | 2916.51 (1015.67, 4817.35) | -848.23 (-1606.59, -89.86) |
| >$731,225 | Ref | Ref |
| Geography |  |  |
| Northeast | -874.30 (-3273.68, 1525.09) | -556.95 (-1510.41, 396.50) |
| Midwest | -429.92 (-2517.95, 1658.12) | -958.83 (-1793.58, -124.08) |
| South | 1426.03 (-476.50, 3328.57) | 276.09 (-488.20, 1040.38) |
| West | Ref | Ref |
| Comorbidities |  |  |
| 0 | -4726.46 (-7047.03, -2405.89) | -2821.95 (-3588.83, -2055.06) |
| 1 | -2021.49 (-3656.32, -386.66) | -2623.78 (-3239.81, -2007.74) |
| ≥2 | Ref | Ref |
| Current smoker |  |  |
| Yes | Ref | Ref |
| No | -717.45 (-3218.88, 1783.98) | -1053.97 (-1896.11, -211.83) |

**Source:** Authors analysis of data from the Health and Retirement Study, 2008-2020.

^a^ Out-of-pocket spending was measured in the survey waves following initial Medicare plan selection and included beneficiaries’ portion of costs for hospital stays, nursing home stays, outpatient surgeries, physician visits, dentist visits, home health care, special services, and prescription medications. Outcomes were pooled and 1,177 and 8,618 observations were included in the models of respondents with and without a history of cancer, respectively.

^b^ Out-of-pocket spending was inflation-adjusted to 2020 dollars using the Consumer Price Index for all Urban Consumers.

**Table S6.** Multivariable Model Assessing the Association of Initial Medicare Plan Choice with Self-reported Out-of-Pocket Spending at the 95^th^ Percentile

|  | **Respondents with a History of Cancer** | **Respondents without a History of Cancer** |
| --- | --- | --- |
|  | **Adjusted Estimates (95% Confidence Limits)**^a,b^ | |
| Intercept | 15267.74 (8605.81, 21929.67) | 15081.21 (12526.07, 17636.36) |
| Initial plan type |  |  |
| Medicare Advantage | -9884.24 (-13383.85, -6384.63) | -2362.26 (-3593.39, -1131.14) |
| Traditional Medicare plus supplemental coverage | -9634.94 (-12873.59, -6396.29) | -452.92 (-1557.17, 651.34) |
| Traditional Medicare without supplemental coverage | Ref | Ref |
| Sex |  |  |
| Male | Ref | Ref |
| Female | -724.41 (-2800.34, 1351.51) | -391.36 (-1245.20, 462.48) |
| Race |  |  |
| White | Ref | Ref |
| Black | -3968.10 (-7125.46, -810.75) | -2509.26 (-3738.30, -1280.23) |
| Other | 5154.97 (-779.33, 11089.27) | 3715.25 (1824.20, 5606.29) |
| Ethnicity |  |  |
| Non-Hispanic | 8328.20 (3702.94, 12953.46) | 1141.32 (-463.84, 2746.49) |
| Hispanic | Ref | Ref |
| Married or partnered |  |  |
| Yes | Ref | Ref |
| No | 1007.90 (-1385.43, 3401.23) | -439.07 (-1438.77, 560.63) |
| Education |  |  |
| High school or less | Ref | Ref |
| Above high school | -1387.51 (-3398.60, 623.57) | 781.10 (-139.22, 1701.43) |
| Wealth |  |  |
| <$84,500 | -1569.46 (-4491.06, 1352.15) | -3241.90 (-4609.26, -1874.53) |
| $84,500-$293,000 | -3222.34 (-6025.30, -419.38) | -1493.73 (-2717.63, -269.84) |
| $293,001-$731,225 | 1785.56 (-812.56, 4383.67) | -1689.10 (-2849.35, -528.85) |
| >$731,225 | Ref | Ref |
| Geography |  |  |
| Northeast | 2812.03 (-467.51, 6091.57) | -879.10 (-2337.83, 579.62) |
| Midwest | -16.57 (-2870.54, 2837.40) | -760.21 (-2037.33, 516.91) |
| South | 3891.93 (1291.50, 6492.36) | 1746.76 (577.44, 2916.08) |
| West | Ref | Ref |
| Comorbidities |  |  |
| 0 | -6769.00 (-9940.82, -3597.19) | -3725.60 (-4898.89, -2552.31) |
| 1 | -3218.93 (05453.56, -984.40) | -3511.71 (-4454.20, -2569.22) |
| ≥2 | Ref | Ref |
| Current smoker |  |  |
| Yes | Ref | Ref |
| No | -694.04 (-4113.06, 2724.97) | -1975.81 (-3624.23, -687.39) |

**Source:** Authors analysis of data from the Health and Retirement Study, 2008-2020.

^a^ Out-of-pocket spending was measured in the survey waves following initial Medicare plan selection and included beneficiaries’ portion of costs for hospital stays, nursing home stays, outpatient surgeries, physician visits, dentist visits, home health care, special services, and prescription medications. Outcomes were pooled and 1,177 and 8,618 observations were included in the models of respondents with and without a history of cancer, respectively.

^b^ Out-of-pocket spending was inflation-adjusted to 2020 dollars using the Consumer Price Index for all Urban Consumers.

**Table S7.** Multivariable Model Assessing the Association of Initial Medicare Plan Choice with Self-reported Cost-related Medication Nonadherence

|  | **Respondents with a History of Cancer** | **Respondents without a History of Cancer** | **Respondents with a History of Cancer** | **Respondents without a History of Cancer** |
| --- | --- | --- | --- | --- |
|  | **aRD (95% CL)** | | **aRR (95% CL)** | |
| Initial plan type |  |  |  |  |
| Medicare Advantage | -0.07 (-0.13, -0.01) | -0.02 (-0.04, 0.01) | 0.33 (0.14, 0.78) | 0.79 (0.59, 1.06) |
| Traditional Medicare plus supplemental coverage | -0.05 (-0.11, 0.02) | -0.01 (-0.04, 0.01) | 0.58 (0.29, 1.16) | 0.82 (0.63, 1.08) |
| Traditional Medicare w/o supplemental coverage | Ref | Ref | Ref | Ref |
| Sex |  |  |  |  |
| Male | Ref | Ref | Ref | Ref |
| Female | 0.03 (-0.004, 0.06) | 0.02 (0.01, 0.04) | 1.69 (0.87, 3.31) | 1.45 (1.15, 1.83) |
| Race |  |  |  |  |
| White | Ref | Ref | Ref | Ref |
| Black | -0.05 (-0.09, -0.01) | 0.01 (-0.02, 0.04) | 0.42 (0.15, 1.20) | 1.15 (0.85, 1.56) |
| Other | 0.06 (-0.06, 0.19) | 0.02 (-0.02, 0.06) | 2.48 (0.70, 8.74) | 1.21 (0.76, 1.93) |
| Ethnicity |  |  |  |  |
| Non-Hispanic | 0.03 (-0.09, 0.14) | 0.0001 (-0.03, 0.03) | 1.53 (0.31, 7.50) | 0.98 (0.67, 1.44) |
| Hispanic | Ref | Ref | Ref | Ref |
| Married or partnered |  |  |  |  |
| Yes | Ref | Ref | Ref | Ref |
| No | 0.01 (-0.03, 0.06) | -0.004 (-0.02, 0.01) | 1.16 (0.63, 2.16) | 0.94 (0.74, 1.18) |
| Education |  |  |  |  |
| High school or less | Ref | Ref | Ref | Ref |
| Above high school | 0.003 (-0.03, 0.04) | -0.01 (-0.02, 0.01) | 1.18 (0.62, 2.24) | 0.85 (0.65, 1.21) |
| Wealth |  |  |  |  |
| <$84,500 | 0.05 (-0.01, 0.11) | 0.09 (0.06, 0.11) | 2.45 (0.88, 6.80) | 5.16 (3.24, 8.22) |
| $84,500-$293,000 | 0.02 (-0.02, 0.07) | 0.05 (0.03, 0.07) | 1.71 (0.66, 4.46) | 3.69 (2.35, 5.79) |
| $293,001-$731,225 | 0.003 (-0.03, 0.04) | 0.01 (-0.0001, 0.02) | 1.03 (0.40, 2.67) | 1.90 (1.18, 3.07) |
| >$731,225 | Ref | Ref | Ref | Ref |
| Geography |  |  |  |  |
| Northeast | 0.04 (-0.02, 0.10) | -0.01 (-0.03, 0.01) | 2.52 (0.80, 7.89) | 0.82 (0.55, 1.22) |
| Midwest | 0.01 (-0.03, 0.05) | -0.01 (-0.03, 0.02) | 1.37 (0.48, 3.91) | 0.88 (0.62, 1.25) |
| South | 0.03 (-0.005, 0.07) | -0.01 (-0.03, 0.01) | 1.98 (0.78, 5.05) | 0.85 (0.63, 1.16) |
| West | Ref | Ref | Ref | Ref |
| Comorbidities |  |  |  |  |
| 0 | -0.03 (-0.06, 0.01) | -0.05 (-0.07, -0.04) | 0.49 (0.15, 1.66) | 0.29 (0.19, 0.45) |
| 1 | -0.01 (-0.05, 0.02) | -0.04 (-0.06, -0.03) | 0.78 (0.36, 1.66) | 0.49 (0.38, 0.65) |
| ≥2 | Ref | Ref | Ref | Ref |
| Current smoker |  |  |  |  |
| Yes | Ref | Ref | Ref | Ref |
| No | -0.04 (-0.11, 0.03) | -0.005 (-0.03, 0.02) | 0.63 (0.31, 1.30) | 0.93 (0.70, 1.24) |

**Source:** Authors’ analysis of data from the Health and Retirement Study, 2008-2020.

**Abbreviations:** aRD, adjusted risk difference; aRR, adjusted risk ratio; CL, confidence limit

^a^ Outcomes were measured in the survey waves following initial Medicare plan selection and were pooled. 1,177 and 8,618 observations were included in the models of respondents with and without a history of cancer, respectively.

^b^ 40 (4 cancer; 36 noncancer) missing observations were excluded from the cost-related nonadherence model.

**Table S8.** Multivariable Model Assessing the Association of Initial Medicare Plan Choice with Any Self-reported Overnight Hospital Stay

|  | **Respondents with a History of Cancer** | **Respondents without a History of Cancer** | **Respondents with a History of Cancer** | **Respondents without a History of Cancer** |
| --- | --- | --- | --- | --- |
|  | **aRDs (95% CLs)** | | **aRRs (95% CLs)** | |
| Initial plan type |  |  |  |  |
| Medicare Advantage | -0.02 (-0.12, 0.09) | -0.02 (-0.06, 0.01) | 0.93 (0.65, 1.32) | 0.89 (0.77, 1.04) |
| Traditional Medicare plus supplemental coverage | 0.03 (-0.07, 0.13) | 0.002 (-0.03, 0.03) | 1.11 (0.82, 1.52) | 1.01 (0.89, 1.15) |
| Traditional Medicare w/o supplemental coverage | Ref | Ref | Ref | Ref |
| Sex |  |  |  |  |
| Male | Ref | Ref | Ref | Ref |
| Female | -0.03 (-0.10, 0.03) | -0.02 (-0.04, 0.002) | 0.86 (0.68, 1.10) | 0.91 (0.83, 1.01) |
| Race |  |  |  |  |
| White | Ref | Ref | Ref | Ref |
| Black | -0.07 (-0.18, 0.04) | -0.01 (-0.05, 0.02) | 0.81 (0.54, 1.20) | 0.94 (0.82, 1.09) |
| Other | -0.07 (-0.28, 0.14) | 0.03 (-0.01, 0.08) | 0.79 (0.33, 1.87) | 1.17 (0.95, 1.44) |
| Ethnicity |  |  |  |  |
| Non-Hispanic | -0.04 (-0.19, 0.11) | 0.08 (0.04, 0.12) | 0.88 (0.55, 1.41) | 1.50 (1.21, 1.87) |
| Hispanic | Ref | Ref | Ref | Ref |
| Married or partnered |  |  |  |  |
| Yes | Ref | Ref | Ref | Ref |
| No | 0.08 (0.002, 0.17) | -0.004 (-0.03, 0.02) | 1.32 (1.03, 1.70) | 0.99 (0.88, 1.10) |
| Education |  |  |  |  |
| High school or less | Ref | Ref | Ref | Ref |
| Above high school | 0.01 (-0.06, 0.07) | -0.02 (-0.05, 0.001) | 1.05 (0.83, 1.32) | 0.90 (0.81, 1.01) |
| Wealth |  |  |  |  |
| <$84,500 | 0.08 (-0.02, 0.17) | 0.02 (-0.01, 0.06) | 1.38 (0.98, 1.94) | 1.13 (0.97, 1.33) |
| $84,500-$293,000 | 0.11 (0.02, 0.21) | 0.03 (-0.01, 0.06) | 1.52 (1.09, 2.14) | 1.14 (0.98, 1.32) |
| $293,001-$731,225 | 0.07 (-0.004, 0.14) | 0.03 (-0.0002, 0.06) | 1.35 (0.98, 1.85) | 1.15 (1.00, 1.33) |
| >$731,225 | Ref | Ref | Ref | Ref |
| Geography |  |  |  |  |
| Northeast | -0.03 (-0.12, 0.06) | 0.03 (-0.01, 0.06) | 0.91 (0.58, 1.41) | 1.14 (0.96, 1.34) |
| Midwest | 0.05 (-0.04, 0.14) | 0.02 (-0.02, 0.05) | 1.22 (0.86, 1.74) | 1.08 (0.93, 1.26) |
| South | 0.08 (0.001, 0.16) | 0.02 (-0.01, 0.04) | 1.34 (0.99, 1.82) | 1.08 (0.93, 1.24) |
| West | Ref | Ref | Ref | Ref |
| Comorbidities |  |  |  |  |
| 0 | -0.17 (-0.25, -0.09) | -0.15 (-0.17, -0.12) | 0.45 (0.28, 0.73) | 0.45 (0.38, 0.53) |
| 1 | -0.13 (-0.20, -0.06) | -0.08 (-0.11, -0.06) | 0.61 (0.46, 0.82) | 0.70 (0.62, 0.78) |
| ≥2 | Ref | Ref | Ref | Ref |
| Current smoker |  |  |  |  |
| Yes | Ref | Ref | Ref | Ref |
| No | -0.04 (-0.15, 0.08) | -0.06 (-0.10, -0.02) | 0.90 (0.65, 1.25) | 0.79 (0.69, 0.91) |

**Source:** Authors’ analysis of data from the Health and Retirement Study, 2008-2020.

**Abbreviations:** aRD, adjusted risk difference; aRR, adjusted risk ratio; CL, confidence limit

^a^ Outcomes were measured in the survey waves following initial Medicare plan selection and were pooled. 1,177 and 8,618 observations were included in the models of respondents with and without a history of cancer, respectively.

^b^ 37 (cancer: 3; noncancer 34) missing observations were excluded from the hospitalizations model.

**Table S9.** Multivariable Model Assessing the Association of Initial Medicare Plan Choice with Self-reported Fair or Poor Health Status

|  | **Respondents with a History of Cancer** | **Respondents without a History of Cancer** | **Respondents with a History of Cancer** | **Respondents without a History of Cancer** |
| --- | --- | --- | --- | --- |
|  | **aRDs (95% CLs)** | | **aRRs (95% CLs)** | |
| Initial plan type |  |  |  |  |
| Medicare Advantage | -0.15 (-0.29, -0.01) | -0.06 (-0.10, -0.02) | 0.60 (0.39, 0.93) | 0.78 (0.66, 0.92) |
| Traditional Medicare plus supplemental coverage | -0.09 (-0.22, 0.05) | -0.04 (-0.08, -0.01) | 0.79 (0.55, 1.15) | 0.83 (0.72, 0.97) |
| Traditional Medicare w/o supplemental coverage | Ref | Ref | Ref | Ref |
| Sex |  |  |  |  |
| Male | Ref | Ref | Ref | Ref |
| Female | -0.01 (-0.08, 0.07) | -0.02 (-0.04, 0.01) | 0.92 (0.67, 1.25) | 0.91 (0.80, 1.04) |
| Race |  |  |  |  |
| White | Ref | Ref | Ref | Ref |
| Black | 0.05 (-0.08, 0.19) | -0.03 (-0.07, 0.01) | 1.23 (0.84, 1.80) | 0.90 (0.75, 1.08) |
| Other | 0.09 (-0.16, 0.35) | 0.09 (0.03, 0.16) | 1.39 (0.61, 3.15) | 1.38 (1.12, 1.69) |
| Ethnicity |  |  |  |  |
| Non-Hispanic | -0.03 (-0.24, 0.17) | -0.11 (-0.16, -0.05) | 0.88 (0.51, 1.55) | 0.69 (0.57, 0.82) |
| Hispanic | Ref | Ref | Ref | Ref |
| Married or partnered |  |  |  |  |
| Yes | Ref | Ref | Ref | Ref |
| No | -0.03 (-0.12, 0.06) | -0.004 (-0.03, 0.03) | 0.89 (0.65, 1.23) | 0.98 (0.85, 1.14) |
| Education |  |  |  |  |
| High school or less | Ref | Ref | Ref | Ref |
| Above high school | -0.12 (-0.19, -0.04) | -0.05 (-0.07, -0.03) | 0.59 (0.42, 0.84) | 0.73 (0.62, 0.86) |
| Wealth |  |  |  |  |
| <$84,500 | 0.10 (-0.01, 0.22) | 0.13 (0.09, 0.18) | 1.43 (0.91, 2.25) | 1.94 (1.55, 2.43) |
| $84,500-$293,000 | 0.02 (-0.08, 0.13) | 0.03 (-0.004, 0.06) | 1.13 (0.70, 1.83) | 1.33 (1.06, 1.66) |
| $293,001-$731,225 | 0.001 (-0.09, 0.09) | 0.004 (-0.02, 0.03) | 1.02 (0.63, 1.66) | 1.13 (0.90, 1.41) |
| >$731,225 | Ref | Ref | Ref | Ref |
| Geography |  |  |  |  |
| Northeast | -0.03 (-0.14, 0.08) | 0.01 (-0.04, 0.05) | 0.89 (0.51, 1.56) | 1.01 (0.82, 1.26) |
| Midwest | -0.04 (-0.15, 0.07) | -0.03 (-0.06, 0.01) | 0.83 (0.51, 1.35) | 0.85 (0.70, 1.04) |
| South | 0.02 (-0.07, 0.12) | -0.003 (-0.04, 0.03) | 1.07 (0.71, 1.60) | 0.96 (0.80, 1.14) |
| West | Ref | Ref | Ref | Ref |
| Comorbidities |  |  |  |  |
| 0 | -0.18 (-0.27, -0.08) | -0.16 (-0.19, -0.13) | 0.34 (0.14, 0.82) | 0.34 (0.27, 0.44) |
| 1 | -0.15 (-0.22, -0.07) | -0.13 (-0.16, -0.10) | 0.49 (0.32, 0.75) | 0.48 (0.41, 0.57) |
| ≥2 | Ref | Ref | Ref | Ref |
| Current smoker |  |  |  |  |
| Yes | Ref | Ref | Ref | Ref |
| No | -0.15 (-0.30, -0.01) | -0.13 (-0.17, -0.08) | 0.64 (0.45, 0.90) | 0.61 (0.52, 0.70) |

**Source:** Authors’ analysis of data from the Health and Retirement Study, 2008-2020.

**Abbreviations:** aRD, adjusted risk difference; aRR, adjusted risk ratio; CL, confidence limit

^a^ Outcomes were measured in the survey waves following initial Medicare plan selection and were pooled. 1,177 and 8,618 observations were included in the models of respondents with and without a history of cancer, respectively.

^b^ 8 (2 cancer; 6 noncancer) missing observations were excluded from the health status model.

**Table S10.** Sensitivity Analysis Assessing the Association of Initial Medicare Plan Choice with Self-reported Financial and Health Burden

|  | **Study Cohort** | **Respondents with a History of Cancer** | **Respondents without a History of Cancer** | **Study Cohort** | **Respondents with a History of Cancer** | **Respondents without a History of Cancer** |
| --- | --- | --- | --- | --- | --- | --- |
|  | **Adjusted RDs (95% CLs)** | | | **Adjusted RRs (95% CLs)** | | |
| Assessed beneficiaries 65-75 years of age at initial plan selection^a^ | | | | | | |
| *Cost-related medication nonadherence* | | | | | | |
| Medicare Advantage | -0.02  (-0.04, -0.002) | -0.08  (-0.15, -0.02) | -0.01  (-0.03, 0.01) | 0.77  (0.61, 0.97) | 0.32  (0.14, 0.72) | 0.84  (0.66, 1.07) |
| Traditional Medicare plus supplemental coverage | -0.02  (-0.04, -0.002) | -0.06  (-0.13, -0.001) | -0.02  (-0.04, 0.001) | 0.77  (0.62, 0.97) | 0.52  (0.28, 0.97) | 0.80  (0.63, 1.01) |
| Traditional Medicare w/o supplemental coverage | Ref | Ref | Ref | Ref | Ref | Ref |
| *Any hospitalization* | | | | | | |
| Medicare Advantage | -0.01  (-0.04, 0.01) | -0.01  (-0.11, 0.09) | -0.01  (-0.04, 0.01) | 0.94  (0.84, 1.06) | 0.95  (0.69, 1.32) | 0.94  (0.83, 1.07) |
| Traditional Medicare plus supplemental coverage | 0.0003  (-0.02, 0.03) | 0.001  (-0.09, 0.09) | 0.001  (-0.03, 0.03) | 1.00  (0.90, 1.11) | 1.01  (0.75, 1.36) | 1.00  (0.90, 1.12) |
| Traditional Medicare w/o supplemental coverage | Ref | Ref | Ref | Ref | Ref | Ref |
| *Fair or poor health status* | | | | | | |
| Medicare Advantage | -0.05  (-0.09, -0.02) | -0.07  (-0.18, 0.04) | -0.05  (-0.09, -0.02) | 0.81  (0.71, 0.92) | 0.79  (0.56, 1.13) | 0.81  (0.71, 0.94) |
| Traditional Medicare plus supplemental coverage | -0.04  (-0.07, -0.02) | -0.03  (-0.14, 0.08) | -0.05  (-0.08, -0.02) | 0.83  (0.74, 0.94) | 0.92  (0.66, 1.27) | 0.82  (0.72, 0.94) |
| Traditional Medicare w/o supplemental coverage | Ref | Ref | Ref | Ref | Ref | Ref |
| Excluded incident cancer cases after initial plan selection^b^ | | | | | | |
| *Cost-related medication nonadherence* | | | | | | |
| Medicare Advantage | -0.01  (-0.04, 0.01) | -0.07  (-0.13, -0.01) | -0.01  (-0.03, 0.02) | 0.81  (0.61, 1.09) | 0.33  (0.14, 0.78) | 0.90  (0.66, 1.22) |
| Traditional Medicare plus supplemental coverage | -0.01  (-0.03, 0.01) | -0.05  (-0.11, 0.02) | -0.01  (-0.03, 0.01) | 0.86  (0.65, 1.12) | 0.58  (0.29, 1.16) | 0.88  (0.66, 1.18) |
| Traditional Medicare w/o supplemental coverage | Ref | Ref | Ref | Ref | Ref | Ref |
| *Any hospitalization* | | | | | | |
| Medicare Advantage | -0.03  (-0.06, 0.005) | -0.02  (-0.12, 0.09) | -0.03  (-0.06, 0.01) | 0.87  (0.75, 1.02) | 0.93  (0.65, 1.32) | 0.87  (0.74, 1.03) |
| Traditional Medicare plus supplemental coverage | 0.01  (-0.02, 0.04) | 0.03  (-0.07, 0.13) | 0.01  (-0.02, 0.04) | 1.04  (0.91, 1.19) | 1.11  (0.82, 1.52) | 1.04  (0.90, 1.21) |
| Traditional Medicare w/o supplemental coverage | Ref | Ref | Ref | Ref | Ref | Ref |
| *Fair or poor health status* | | | | | | |
| Medicare Advantage | -0.06  (-0.10, -0.03) | -0.15  (-0.29, -0.01) | -0.05  (-0.09, -0.01) | 0.74  (0.63, 0.88) | 0.60  (0.39, 0.93) | 0.78  (0.65, 0.94) |
| Traditional Medicare plus supplemental coverage | -0.04  (-0.07, -0.001) | -0.09  (-0.22, 0.05) | -0.03  (-0.07, 0.005) | 0.86  (0.74, 1.00) | 0.79  (0.55, 1.15) | 0.87  (0.74, 1.02) |
| Traditional Medicare w/o supplemental coverage | Ref | Ref | Ref | Ref | Ref | Ref |
| Excluded respondents who switched Medicare plans^c^ | | | | | | |
| *Cost-related medication nonadherence* | | | | | | |
| Medicare Advantage | -0.02  (-0.05, 0.003) | -0.05  (-0.13, 0.03) | -0.02  (-0.05, 0.01) | 0.72  (0.51, 1.03) | 0.45  (0.13, 1.58) | 0.78  (0.54, 1.12) |
| Traditional Medicare plus supplemental coverage | -0.02  (-0.04, 0.01) | -0.03  (-0.11, 0.05) | -0.01  (-0.04, 0.01) | 0.82  (0.59, 1.14) | 0.71  (0.27, 1.90) | 0.84  (0.60, 1.19) |
| Traditional Medicare w/o supplemental coverage | Ref | Ref | Ref | Ref | Ref | Ref |
| *Any hospitalization* | | | | | | |
| Medicare Advantage | -0.02  (-0.06, 0.02) | -0.04  (-0.20, 0.13) | -0.02  (-0.06, 0.02) | 0.91  (0.77, 1.09) | 0.91  (0.57, 1.44) | 0.92  (0.76, 1.11) |
| Traditional Medicare plus supplemental coverage | 0.02  (-0.02, 0.06) | -0.003  (-0.16, 0.16) | 0.02  (-0.02, 0.06) | 1.09  (0.93, 1.27) | 1.05  (0.70, 1.59) | 1.09  (0.93, 1.29) |
| Traditional Medicare w/o supplemental coverage | Ref | Ref | Ref | Ref | Ref | Ref |
| *Fair or poor health status* | | | | | | |
| Medicare Advantage | -0.08  (-0.12, -0.03) | -0.14  (-0.34, 0.06) | -0.08  (-0.12, -0.02) | 0.74  (0.61, 0.89) | 0.64  (0.37, 1.13) | 0.76  (0.62, 0.93) |
| Traditional Medicare plus supplemental coverage | -0.05  (-0.10, -0.01) | -0.08  (-0.27, 0.12) | -0.05  (-0.10, -0.01) | 0.82  (0.69, 0.97) | 0.84  (0.51, 1.39) | 0.82  (0.68, 0.98) |
| Traditional Medicare w/o supplemental coverage | Ref | Ref | Ref | Ref | Ref | Ref |
| Controlled for baseline outcomes in the models^d^ | | | | | | |
| *Cost-related medication nonadherence* | | | | | | |
| Medicare Advantage | -0.02  (-0.04, 0.003) | -0.05  (-0.13, 0.03) | -0.02  (-0.05, 0.01) | 0.78  (0.60, 1.01) | 0.30  (0.12, 0.72) | 0.87  (0.66, 1.14) |
| Traditional Medicare plus supplemental coverage | -0.01  (-0.03, 0.01) | -0.03  (-0.11, 0.05) | -0.01  (-0.04, 0.01) | 0.87  (0.68, 1.10) | 0.60  (0.30, 1.23) | 0.89  (0.69, 1.14) |
| Traditional Medicare w/o supplemental coverage | Ref | Ref | Ref | Ref | Ref | Ref |
| *Any hospitalization* | | | | | | |
| Medicare Advantage | -0.02  (-0.05, 0.01) | -0.03  (-0.13, 0.07) | -0.02  (-0.05, 0.01) | 0.90  (0.79, 1.04) | 0.89  (0.63, 1.26) | 0.91  (0.79, 1.06) |
| Traditional Medicare plus supplemental coverage | 0.001  (-0.03, 0.03) | 0.03  (-0.07, 0.12) | -0.001  (-0.03, 0.03) | 1.01  (0.90, 1.13) | 1.12  (0.82, 1.52) | 1.00  (0.88, 1.13) |
| Traditional Medicare w/o supplemental coverage | Ref | Ref | Ref | Ref | Ref | Ref |
| *Fair or poor health status* | | | | | | |
| Medicare Advantage | -0.04  (-0.07, -0.004) | -0.02  (-0.13, 0.09) | -0.03  (-0.07, -0.001) | 0.88  (0.76, 1.01) | 0.94  (0.65, 1.38) | 0.88  (0.76, 1.02) |
| Traditional Medicare plus supplemental coverage | -0.02  (-0.05, 0.01) | 0.03  (-0.07, 0.13) | -0.03  (-0.06, 002) | 0.92  (0.81, 1.04) | 1.23  (0.87, 1.72) | 0.89  (0.78, 1.03) |
| Traditional Medicare w/o supplemental coverage | Ref | Ref | Ref | Ref | Ref | Ref |
| Removed wealth and assets from the models^d^ | | | | | | |
| *Cost-related medication nonadherence* | | | | | | |
| Medicare Advantage | -0.03  (-0.05, -0.01) | -0.07  (-0.13, -0.01) | -0.02  (-0.05, -0.01) | 0.69  (0.52, 0.92) | 0.33  (0.14, 0.76) | 0.74  (0.55, 0.99) |
| Traditional Medicare plus supplemental coverage | -0.03  (-0.05, -0.01) | -0.05  (-0.11, 0.01) | -0.03  (-0.05, -0.004) | 0.70  (0.54, 0.91) | 0.51  (0.26, 0.98) | 0.71  (0.54, 0.94) |
| Traditional Medicare w/o supplemental coverage | Ref | Ref | Ref | Ref | Ref | Ref |

**Source:** Authors’ analysis of data from the Health and Retirement Study, 2008-2020.

**Abbreviations:** CL, confidence limit; RD, risk difference; RR, risk ratio

^a^ Analysis included beneficiaries who were 65 to 75 years of age at initial Medicare plan selection. Outcomes were measured in the survey waves following initial Medicare plan selection and were pooled. 1,508 and 10,906 observations were included in the models of respondents with and without a history of cancer, respectively. Missing observations were excluded from the models. Specifically, 53 (4 cancer; 49 noncancer) observations were excluded from the cost-related medication nonadherence model; 46 (4 cancer; 42 noncancer) observations were excluded from the hospitalizations model; and 13 (3 cancer; 10 noncancer) observations were excluded from the health status model.

^b^ Analysis excluded beneficiaries who reported a cancer diagnosis in the survey waves after initial Medicare plan selection. Outcomes were measured in the survey waves following initial Medicare plan selection and were pooled. 1,177 and 7,576 observations were included in the models of respondents with and without a history of cancer, respectively. Missing observations were excluded from the models. Specifically, 38 (4 cancer; 34 noncancer) observations were excluded from the cost-related medication nonadherence model; 36 (3 cancer; 33 noncancer) observations were excluded from the hospitalizations model; and 7 (2 cancer; 5 noncancer) observations were excluded from the health status model.

^c^ Analysis excluded beneficiaries who reported switching Medicare plans in the survey immediately after initial Medicare plan selection. Outcomes were measured in the survey waves following initial Medicare plan selection and were pooled. 849 and 6,170 observations were included in the models of respondents with and without a history of cancer, respectively. Missing observations were excluded from the models. Specifically, 33 (1 cancer; 32 noncancer) observations were excluded from the cost-related medication nonadherence model; 36 (4 cancer; 32 noncancer) observations were excluded from the hospitalizations model; and 6 (2 cancer; 4 noncancer) observations were excluded from the health status model.

^d^ Outcomes were measured in the survey waves following initial Medicare plan selection and were pooled. 1,177 and 8,618 observations were included in the models of respondents with and without a history of cancer, respectively. Missing observations were excluded from the models. Specifically, 40 (4 cancer; 36 noncancer) observations were excluded from the cost-related medication nonadherence model.

**Table S11.** Sensitivity Analysis Assessing the Association of Initial Medicare Plan Selection with Self-reported Out-of-Pocket Spending (Among Beneficiaries Aged 65-75 at Initial Plan Selection)

|  | **Study Cohort** | **Respondents with a History of Cancer** | **Respondents without a History of Cancer** |
| --- | --- | --- | --- |
| **Adjusted Estimates (95% Confidence Limits)** | | | |
| **25^th^ Percentile** | | | |
| Medicare Advantage | -45.40  (-107.10, 16.30) | -25.63  (-236.18, 184.91) | -36.39  (-101.77, 28.99) |
| Traditional Medicare plus supplemental coverage | 43.41  (-12.83, 99.64) | 160.30  (-38.43, 359.03) | 45.47  (-13.92, 104.85) |
| Traditional Medicare w/o supplemental coverage | Ref | Ref | Ref |
| **50^th^ Percentile** | | | |
| Medicare Advantage | -193.01  (-319.33, -66.73) | -198.51  (-690.65, 293.64) | -193.79  (-321.86, -65.71) |
| Traditional Medicare plus supplemental coverage | 48.83  (-66.26, 163.95) | 131.90  (-332.61, 596.42) | 44.01  (-72.32, 160.34) |
| Traditional Medicare w/o supplemental coverage | Ref | Ref | Ref |
| **75^th^ Percentile** | | | |
| Medicare Advantage | -525.64  (-795.01, -256.27) | -758.00  (-1669.56, 153.56) | -497.47  (-785.24, -209.70) |
| Traditional Medicare plus supplemental coverage | 5.98  (-239.55, 251.51) | -334.00  (-1194.38, 526.38) | 44.94  (-216.44, 306.31) |
| Traditional Medicare w/o supplemental coverage | Ref | Ref | Ref |
| **90^th^ Percentile** | | | |
| Medicare Advantage | -1413. 24  (-2095.23, -731.26) | -1820.16  (-3983.48, 343.16) | -1414.68  (-2197.16, -632.20) |
| Traditional Medicare plus supplemental coverage | -477.08  (-1098.71, 144.55) | -2186.68  (-4228.55, -144.80) | -402.96  (-1113.67, 307.75) |
| Traditional Medicare w/o supplemental coverage | Ref | Ref | Ref |
| **95^th^ Percentile** | | | |
| Medicare Advantage | -2130.14  (-3235.04, -1025.24) | -5771.33  (-8994.91, -2547.76) | -2001.51  (-3129.07, -873.96) |
| Traditional Medicare plus supplemental coverage | -429.24  (-1436.36, 577.88) | -6105.43  (-9148.04, -3062.82) | -95.60  (-1119.72, 928.52) |
| Traditional Medicare w/o supplemental coverage | Ref | Ref | Ref |

**Source:** Authors’ analysis of data from the Health and Retirement Study, 2008-2020.

^a^ Table presents adjusted out-of-pocket spending. Analysis included beneficiaries who were 65 to 75 years of age at initial Medicare plan selection.

^b^ Out-of-pocket spending was measured in the survey waves following initial Medicare plan selection and included beneficiaries’ portion of costs for hospital stays, nursing home stays, outpatient surgeries, physician visits, dentist visits, home health care, special services, and prescription medications. Outcomes were pooled and 1,508 and 10,906 observations were included in the models of respondents with and without a history of cancer, respectively.

^c^ Out-of-pocket spending was inflation-adjusted to 2020 dollars using the Consumer Price Index for all Urban Consumers.

**Table S12.** Sensitivity Analysis Assessing the Association of Initial Medicare Plan Selection with Self-reported Out-of-Pocket Spending (Excluding Beneficiaries with a Cancer Diagnosis After Initial Plan Selection)

|  | **Study Cohort** | **Respondents with a History of Cancer** | **Respondents without a History of Cancer** |
| --- | --- | --- | --- |
| **Adjusted Estimates (95% Confidence Limits)** | | | |
| **25^th^ Percentile** | | | |
| Medicare Advantage | -52.93  (-125.88, 20.02) | -303.78  (-567.24, -40.32) | -30.93  (-101.07, 40.29) |
| Traditional Medicare plus supplemental coverage | 27.44  (-38.37, 93.25) | -139.07  (-382.88, 104.75) | 54.41  (-9.08, 117.89) |
| Traditional Medicare w/o supplemental coverage | Ref | Ref | Ref |
| **50^th^ Percentile** | | | |
| Medicare Advantage | -189.43  (-336.93, -41.93) | -668.67  (-1177.90, -159.44) | -165.81  (-315.45, -16.17) |
| Traditional Medicare plus supplemental coverage | 44.45  (-88.61, 177.52) | -232.63  (-703.89, 238.63) | 109.58  (-24.83, 243.99) |
| Traditional Medicare w/o supplemental coverage | Ref | Ref | Ref |
| **75^th^ Percentile** | | | |
| Medicare Advantage | -605.76  (-935.53, -274.99) | -1094.31  (-2053.67, -134.96) | -571.40  (-913.98, -228.82) |
| Traditional Medicare plus supplemental coverage | -13.84  (-311.34, 283.66) | -875.93  (-1763.74, 11.89) | 43.28  (-264.43, 350.99) |
| Traditional Medicare w/o supplemental coverage | Ref | Ref | Ref |
| **90^th^ Percentile** | | | |
| Medicare Advantage | -1226.82  (-1953.39, -500.24) | -2613.55  (-5173.95, -53.16) | -1084.28  (-1866.12, -302.44) |
| Traditional Medicare plus supplemental coverage | -454.03  (-1109.51, 201.44) | -3195.03  (-5564.50, -825.56) | -264.30  (-966.55, 437.96) |
| Traditional Medicare w/o supplemental coverage | Ref | Ref | Ref |
| **95^th^ Percentile** | | | |
| Medicare Advantage | -2231.32  (-3445.60, -1017.04) | -9884.27  (-13383.85, -6384.70) | -2038.93  (-3300.26, -777.60) |
| Traditional Medicare plus supplemental coverage | -174.46  (-1269.92, 921.00) | -9634.96  (-12873.59, -6396.34) | 0.74  (-1132.21, 1133.68) |
| Traditional Medicare w/o supplemental coverage | Ref | Ref | Ref |

**Source:** Authors’ analysis of data from the Health and Retirement Study, 2008-2020.

^a^ Table presents adjusted out-of-pocket spending. Analysis excluded beneficiaries who reported a cancer diagnosis in the survey waves after initial Medicare plan selection.

^b^ Out-of-pocket spending was measured in the survey waves following initial Medicare plan selection and included beneficiaries’ portion of costs for hospital stays, nursing home stays, outpatient surgeries, physician visits, dentist visits, home health care, special services, and prescription medications. Outcomes were pooled and 1,177 and 7,576 observations were included in the models of respondents with and without a history of cancer, respectively.

^c^ Out-of-pocket spending was inflation-adjusted to 2020 dollars using the Consumer Price Index for all Urban Consumers.

**Table S13.** Sensitivity Analysis Assessing the Association of Initial Medicare Plan Selection with Self-reported Out-of-Pocket Spending (Excluded Beneficiaries who Switched Medicare Coverage After Initial Selection)

|  | **Study Cohort** | **Respondents with a History of Cancer** | **Respondents without a History of Cancer** |
| --- | --- | --- | --- |
| **Adjusted Estimates (95% Confidence Limits)** | | | |
| **25^th^ Percentile** | | | |
| Medicare Advantage | 22.60  (-63.84, 109.04) | -105.04  (-488.72, 278.64) | 26.80  (-55.37, 108.97) |
| Traditional Medicare plus supplemental coverage | 153.14  (72.81, 233.47) | 36.02  (-326.75, 398.79) | 166.86  (90.81, 244.91) |
| Traditional Medicare w/o supplemental coverage | Ref | Ref | Ref |
| **50^th^ Percentile** | | | |
| Medicare Advantage | -3.78  (-171.93, 164.37) | -193.47  (-932.78, 545.84) | -21.14  (-189.16, 146.89) |
| Traditional Medicare plus supplemental coverage | 324.62  (168.36, 480.88) | 213.46  (-485.56, 912.48) | 326.73  (171.23, 482.24) |
| Traditional Medicare w/o supplemental coverage | Ref | Ref | Ref |
| **75^th^ Percentile** | | | |
| Medicare Advantage | -397.37  (-774.65, -20.09) | -173.50  (-1575.05, 1228.06) | -401.04  (-782.79, -19.29) |
| Traditional Medicare plus supplemental coverage | 295.69  (-54.91, 646.30) | 74.31  (-1250.86, 1399.48) | 291.75  (-61.56, 645.06) |
| Traditional Medicare w/o supplemental coverage | Ref | Ref | Ref |
| **90^th^ Percentile** | | | |
| Medicare Advantage | -1211.08  (-2058.30, -363.87) | -1100.90  (-4729.20, 2527.40) | -1198.53  (-2082.58, -314.48) |
| Traditional Medicare plus supplemental coverage | -304.04  (-1091.35, 483.27) | -1994.99  (-5425.55, 1435.57) | -187.77  (-1005.95, 630.42) |
| Traditional Medicare w/o supplemental coverage | Ref | Ref | Ref |
| **95^th^ Percentile** | | | |
| Medicare Advantage | -1743.81  (-3370.50, -117.12) | -3802.01  (-9591.70, 1987.69) | -2062.95  (-3574.11, -551.79) |
| Traditional Medicare plus supplemental coverage | -490.99  (-2002.68, 1020.69) | -3497.67  (-8971.83, 1976.49) | -504.59  (-1903.17, 893.98) |
| Traditional Medicare w/o supplemental coverage | Ref | Ref | Ref |

**Source:** Authors’ analysis of data from the Health and Retirement Study, 2008-2020.

^a^ Table presents adjusted out-of-pocket spending. Analysis excluded beneficiaries who switched Medicare plans in the survey wave immediately after initial Medicare plan selection.

^b^ Out-of-pocket spending was measured in the survey waves following initial Medicare plan selection and included beneficiaries’ portion of costs for hospital stays, nursing home stays, outpatient surgeries, physician visits, dentist visits, home health care, special services, and prescription medications. Outcomes were pooled and 849 and 6,170 observations were included in the models of respondents with and without a history of cancer, respectively.

^c^ Out-of-pocket spending was inflation-adjusted to 2020 dollars using the Consumer Price Index for all Urban Consumers.

**Table S14.** Sensitivity Analysis Assessing the Association of Initial Medicare Plan Selection with Self-reported Out-of-Pocket Spending (Adjusted for Baseline Out-of-Pocket Spending)

|  | **Study Cohort** | **Respondents with a History of Cancer** | **Respondents without a History of Cancer** |
| --- | --- | --- | --- |
| **Adjusted Estimates (95% Confidence Limits)** | | | |
| **25^th^ Percentile** | | | |
| Medicare Advantage | -19.95  (-86.64, 46.74) | -65.67  (-301.48, 170.14) | -6.73  (-74.19, 60.74) |
| Traditional Medicare plus supplemental coverage | 33.43  (-26.60, 93.46) | 40.49  (-178.77, 259.76) | 47.05  (-13.44, 107.55) |
| Traditional Medicare w/o supplemental coverage | Ref | Ref | Ref |
| **50^th^ Percentile** | | | |
| Medicare Advantage | -153.98  (-280.63, -27.32) | -664.32  (-1148.63, -180.02) | -142.73  (-276.30, -9.17) |
| Traditional Medicare plus supplemental coverage | 17.20  (-96.81, 131.21) | -226.17  (-676.50, 224.16) | 23.18  (-96.52, 142.95) |
| Traditional Medicare w/o supplemental coverage | Ref | Ref | Ref |
| **75^th^ Percentile** | | | |
| Medicare Advantage | -502.03  (-786.19, -217.87) | -1126.73  (-1908.30, -345.15) | -525.97  (-848.90, -203.04) |
| Traditional Medicare plus supplemental coverage | -7.43  (-263.21, 248.36) | -296.48  (-1023.22, 430.27) | -51.09  (-340.66, 238.47) |
| Traditional Medicare w/o supplemental coverage | Ref | Ref | Ref |
| **90^th^ Percentile** | | | |
| Medicare Advantage | -1367.04  (-2014.31, -719.77) | -1004.47  (-3077.59, 1068.64) | -1210.29  (-1842.15, -578.42) |
| Traditional Medicare plus supplemental coverage | -857.62  (-1440.26, -274.97) | -1317.96  (-3245.63, 609.72) | -559.51  (-1126.10, 7.07) |
| Traditional Medicare w/o supplemental coverage | Ref | Ref | Ref |
| **95^th^ Percentile** | | | |
| Medicare Advantage | -1772.52  (-3011.59, -533.45) | -5157.82  (-7633.92, -2681.72) | -1656.72  (-2943.88, -368.56) |
| Traditional Medicare plus supplemental coverage | -1060.09  (-2175.44, 55.26) | -5878.63  (-8181.01, -3576.24) | -736.35  (-1890.52, 417.83) |
| Traditional Medicare w/o supplemental coverage | Ref | Ref | Ref |

**Source:** Authors’ analysis of data from the Health and Retirement Study, 2008-2020.

^a^ Table presents adjusted out-of-pocket spending. Analysis included baseline measure of out-of-pocket spending

^b^ Out-of-pocket spending was measured in the survey waves following initial Medicare plan selection and included beneficiaries’ portion of costs for hospital stays, nursing home stays, outpatient surgeries, physician visits, dentist visits, home health care, special services, and prescription medications. Outcomes were pooled and 1,177 and 8,618 observations were included in the models of respondents with and without a history of cancer, respectively.

^c^ Out-of-pocket spending was inflation-adjusted to 2020 dollars using the Consumer Price Index for all Urban Consumers.

**Table S15.** Sensitivity Analysis Assessing the Association of Initial Medicare Plan Selection with Self-reported Out-of-Pocket Spending (Excluded Wealth and Assets from the Model)

|  | **Study Cohort** | **Respondents with a History of Cancer** | **Respondents without a History of Cancer** |
| --- | --- | --- | --- |
| **Adjusted Estimates (95% Confidence Limits)** | | | |
| **25^th^ Percentile** | | | |
| Medicare Advantage | -28.12  (-93.12, 36.88) | -302.11  (-570.59, -33.62) | -11.01  (-77.59, 55.57) |
| Traditional Medicare plus supplemental coverage | 59.32  (1.18, 117.46) | -153.60  (-401.35, 94.15) | 84.92  (25.58, 144.26) |
| Traditional Medicare w/o supplemental coverage | Ref | Ref | Ref |
| **50^th^ Percentile** | | | |
| Medicare Advantage | -203.42  (-350.16, -56.69) | -776.37  (-1291.27, -261.47) | -177.36  (-332.68, -22.04) |
| Traditional Medicare plus supplemental coverage | 79.83  (-51.42, 211.07) | -347.40  (-822.54, 127.75) | 110.65  (-27.78, 249.09) |
| Traditional Medicare w/o supplemental coverage | Ref | Ref | Ref |
| **75^th^ Percentile** | | | |
| Medicare Advantage | -514.80  (-828.37, -201.23) | -856.46  (-1822.12, 109.21) | -484.24  (-805.73, -162.75) |
| Traditional Medicare plus supplemental coverage | 23.59  (-256.88, 304.06) | -584.37  (-1475.47, 306.73) | 121.14  (-165.40, 407.69) |
| Traditional Medicare w/o supplemental coverage | Ref | Ref | Ref |
| **90^th^ Percentile** | | | |
| Medicare Advantage | -1157.67  (-1916.87, -398.46) | -2578.54  (-5300.87, 143.78) | -1123.17  (-1898.80, -347.53) |
| Traditional Medicare plus supplemental coverage | -313.00  (-992.07, 366.07) | -2901.23  (-5413.35, -389.11) | -248.81  (-940.12, 442.51) |
| Traditional Medicare w/o supplemental coverage | Ref | Ref | Ref |
| **95^th^ Percentile** | | | |
| Medicare Advantage | -2153.90  (-3445.59, -862.21) | -8166.74  (-10784.34, -5553.14) | -1985.12  (-3316.31, -653.92) |
| Traditional Medicare plus supplemental coverage | -439.30  (-1594.65, 716.05) | -7625.65  (-10039.29, -5212.02) | -137.09  (-1323.58, 1049.39) |
| Traditional Medicare w/o supplemental coverage | Ref | Ref | Ref |

**Source:** Authors’ analysis of data from the Health and Retirement Study, 2008-2020.

^a^ Table presents adjusted out-of-pocket spending. Analysis excluded wealth and assets from the multivariable model.

^b^ Out-of-pocket spending was measured in the survey waves following initial Medicare plan selection and included beneficiaries’ portion of costs for hospital stays, nursing home stays, outpatient surgeries, physician visits, dentist visits, home health care, special services, and prescription medications. Outcomes were pooled and 1,177 and 8,618 observations were included in the models of respondents with and without a history of cancer, respectively.

^c^ Out-of-pocket spending was inflation-adjusted to 2020 dollars using the Consumer Price Index for all Urban Consumers.
